# Non-invasive measurements of respiration and heart rate across wildlife species using Eulerian Video Magnification of infrared thermal imagery

**DOI:** 10.1186/s12915-023-01555-9

**Published:** 2023-03-29

**Authors:** Caroline L. Rzucidlo, Erin Curry, Michelle R. Shero

**Affiliations:** 1 MIT-WHOI Joint Program in Oceanography/Applied Ocean Science & Engineering, Woods Hole and Cambridge, MA USA; 2 Biology Department, Woods Hole Oceanographic Institution, Woods Hole, MA USA; 3 Center for Conservation and Research of Endangered Wildlife (CREW), Cincinnati Zoo & Botanical Garden, Cincinnati, OH USA

**Keywords:** Conservation, Remote monitoring, Animal health, Veterinary technologies, Metabolic rate

## Abstract

**Background:**

An animal’s metabolic rate, or energetic expenditure, both impacts and is impacted by interactions with its environment. However, techniques for obtaining measurements of metabolic rate are invasive, logistically difficult, and costly. Red–green–blue (RGB) imaging tools have been used in humans and select domestic mammals to accurately measure heart and respiration rate, as proxies of metabolic rate. The purpose of this study was to investigate if infrared thermography (IRT) coupled with Eulerian video magnification (EVM) would extend the applicability of imaging tools towards measuring vital rates in exotic wildlife species with different physical attributes.

**Results:**

We collected IRT and RGB video of 52 total species (39 mammalian, 7 avian, 6 reptilian) from 36 taxonomic families at zoological institutions and used EVM to amplify subtle changes in temperature associated with blood flow for respiration and heart rate measurements. IRT-derived respiration and heart rates were compared to ‘true’ measurements determined simultaneously by expansion of the ribcage/nostrils and stethoscope readings, respectively. Sufficient temporal signals were extracted for measures of respiration rate in 36 species (85% success in mammals; 50% success in birds; 100% success in reptiles) and heart rate in 24 species (67% success in mammals; 33% success in birds; 0% success in reptiles) using IRT-EVM. Infrared-derived measurements were obtained with high accuracy (respiration rate, mean absolute error: 1.9 breaths per minute, average percent error: 4.4%; heart rate, mean absolute error: 2.6 beats per minute, average percent error: 1.3%). Thick integument and animal movement most significantly hindered successful validation.

**Conclusion:**

The combination of IRT with EVM analysis provides a non-invasive method to assess individual animal health in zoos, with great potential to monitor wildlife metabolic indices in situ.

**Supplementary Information:**

The online version contains supplementary material available at 10.1186/s12915-023-01555-9.

## Background

An animal’s survival and reproductive success depends on the individual’s ability to efficiently manage energy stores despite large scale intra- and inter- annual environmental variation [1]. Metabolic rate is modulated by intrinsic factors such as body size, activity levels, and shifts in circulating hormone levels, as well as extrinsic environmental variables that influence thermoregulatory costs, and/or prey and resource availability [2]. Traditional methods for measuring metabolic rate (directly or indirectly) through respirometry or isotope dilution techniques are invasive, logistically difficult, require large time commitments, and are costly. Simpler indices such as heart rate (HR) [3, 4] and respiration rate (RR) [5] that are tightly correlated with energetic expenditure across taxa are thus used as common indices of animal health. Even so, the use of simpler metrics (RR and HR) as proxies of energetic expenditure in wild animals still requires that animals be physically or chemically immobilized [6–8], which comes with inherent risk. Advances in the biotelemetry field allow for longer HR records in free ranging animals through loggers attached to the skin or surgically implanted, however these too are expensive and can be invasive [6], making them impractical for widespread monitoring. Obtaining vital sign measurements using non-invasive imagery would broaden the reach of monitoring efforts, but methodological development is necessary to discern subtle physiological signals of interest to make such animal health assessments possible.

Technological and analytical advancements provide promising avenues for application to wildlife and exotic species. For example, Eulerian video magnification (EVM) can be used to amplify and visualize subtle signals. First, a spatial decomposition is applied to videos such that variation in pixel color at a given location can then be amplified using temporal filtering [9]. EVM of red–green–blue (RGB) video has been successfully used to measure HR in humans [9], primates [10], axolotls, and zebrafish [11]; all have large areas of the body without fur or are translucent.

Diagnostic tools have also been developed using infrared thermography (IRT), the measurement of infrared radiation emitted from an object to capture thermal information. In humans, IRT has been used to screen travelers for fever [12], identify physiological distress via breathing dynamics [13], and accurately measure heart rate [14]. IRT has also been used as a non-invasive tool to promote livestock welfare by detecting lesions and fever in pigs [15] and recognizing increased temperature associated with stress or disease in the dairy and beef industries [16–18]. As the implementation of IRT has gained popularity, it has been useful in measuring a broad range of physiological variables in animals such as shifts in body temperature associated with reproduction [19], hibernation [20, 21], disease [22], and heat flux [23, 24]. IRT has been used to monitor animal vital signs during immobilization by measuring body temperature in dogs [25] and wearable near-IR spectroscopy devices used to measure oxygen saturation in seals [26]. IRT has also been used to monitor vital rates in cetaceans in the wild [27], however because the animals were free-living, these measurements could not be validated. There is also a growing list of studies that use IRT to measure body temperature across taxa (reviewed in [28, 29]).

Although the use of non-contact IRT imaging for obtaining physiological measurements in humans and livestock is well documented, its use in zoo animals and wildlife is primarily limited to the measurement of body temperature and it has yet to be determined whether vital sign measurements could be readily translated across taxa. IRT may make fine-scale changes in heat associated with pulsation more apparent in non-domestic and exotic species that have subcutaneous fat or a thick pelage that would otherwise obscure fluctuations in skin surface coloration by RGB imaging. In this study, we tested whether IRT-derived RR and HR measurements accurately reflected ‘true’ measurements across a range of species, and whether certain physical features of exotic animals (variation in body size and shape, presence of fur/feathers/subcutaneous fat, thick integument) that alter emissivity would impact utility. The addition of EVM processing could make vital sign measurements with IRT broadly applicable across taxa. Confirming that IRT coupled with EVM analysis can accurately describe animal physiological status will allow it to be used as a non-invasive, time efficient method to measure basic metrics of health and metabolism for animals both in human care and in the wild.

## Results

To test whether EVM analysis of IRT imagery can be broadly applied as a non-invasive tool to measure animal vital signs, 58 individuals across 36 families (28 mammals, 4 birds, 4 reptiles) and 52 species (39 mammals, 7 birds, 6 reptiles) were imaged at the Cincinnati Zoo and Botanical Garden in Cincinnati, OH (*n* = 44), the Louisville Zoo in Louisville, KY (*n* = 11), the Columbus Zoo and Aquarium in Columbus, OH (*n* = 2), and the Salisbury Zoo in Salisbury, MD (*n* = 1) (Table 1). Infrared images and videos were recorded using a FLIR T540 camera (30 Hz image frequency, 464 × 348 pixel IR resolution) with a 24° lens (Teledyne FLIR, Wilsonville, OR) placed on a tripod. A GoPro Hero 4 (GoPro, San Mateo, CA) was attached to the tripod and recorded red–green–blue (RGB) color video simultaneously. To determine which point locations on the subjects’ bodies would be most useful for non-invasive vital rate measurements, multiple videos were taken across the body at areas with relatively high thermal signatures and the least amount of movement.

**Table 1:** Species imaged, their physical characteristics, successful RR and HR measurements

Species (common name)	Scientific name	Taxonomic family	Immobilized or voluntary	Color	Thickness of integument or pelage/plumage	Significant subcutaneous fat	Video quality score (out of 8)	True RR	IRT RR	IRT RR location	True HR	IRT HR	IRT HR location
African crested porcupine	*Hystrix cristata*	Hystricidae	voluntary	gray	thick	no	4				120		
Andean bear	*Tremarctos ornatus*	Ursidae	immobilized	black	thick	no	5				40	42	inner leg
Barn owl	*Tyto alba*	Tytonidae	voluntary	tan	thick	no	5						
Bat-eared fox	*Otocyon megalotis*	Canidae	voluntary	brown	thick	no	4						
Blue penguin	*Eudyptula minor*	Spheniscidae	voluntary	black, white	thick	yes	4						
Bonobo	*Pan paniscus*	Hominidae	voluntary	black	thin	no	7	22	20	nostrils, chest	82	77.4	chest, palm
Brown bear	*Ursus arctos*	Ursidae	voluntary	dark brown	thick	yes	6						
California sea lion	*Zalophus californianus*	Otariidae	voluntary	gray	thin	yes	6	18	20	nostrils, abdomen			
Central American tapir	*Tapirus bairdi*	Tapiridae	voluntary	black, white	thin	no	5						
Cheetah	*Acinonyx jubatus*	Felidae	immobilized	tan	thin	no	7	16	15	abdomen, chest	80	84	inner leg
Chuckwalla	*Sauromalus ater*	Iguanidae	voluntary	gray	thick	no	7	22	23	abdomen			
Dabb spiny tailed lizard	*Uromastyx acanthinura*	Agamidae	voluntary	gray	thick	no	5	21	24	neck, nostrils			
Domestic cat	*Felis catus*	Felidae	immobilized	white	thick	no	8	43	44	abdomen	116	112.5	stomach
Dromedary camel	*Camelus dromedarius*	Camelidae	voluntary	tan	thin	no	6	10	12	chest			
Eastern bongo	*Tragelaphus eurycerus*	Bovidae	voluntary	tan	thin	no	8	29	31	abdomen	71	70.2	inner leg
Emu	*Dromaius novaehollandiae*	Dromaiidae	voluntary	brown	thin	no	6	11	7	nostrils	40	42	face
Gopher tortoise	*Gopherus polyphemus*	Testudinidae	voluntary	gray	thick	no	5	24					
Gorilla	*Gorilla gorilla*	Hominidae	immobilized	black	thin	no	7	18	20	nostrils, chest	80	77.4	ear, chest, palm
Gorilla	*Gorilla gorilla*	Hominidae	voluntary	black	thin	no	5	18	18	nostrils, chest	86	91	hand, chest
Gray seal	*Halichoerus grypus*	Phocidae	voluntary	gray	thin	yes	6	40	38	mouth	103	98.4	face
Gray woolly monkey	*Lagothrix cana*	Atelidae	voluntary	brown	thick	no	8	29	29	chest	172	175.8	chest, palm
Harbor seal	*Phoca vitulina*	Phocidae	voluntary	gray	thin	yes	6	14	15	nostrils			
Hippopotamus	*Hippopotamus amphibius*	Hippopotamidae	voluntary	gray	thick	yes	5						
King penguin	*Aptenodytes patagonicus*	Spheniscidae	voluntary	black, white	thick	yes	4						
Lace monitor	*Varanus varius*	Varanidae	voluntary	black, yellow	thick	no	6	44	45	neck			
Lace monitor	*Varanus varius*	Varanidae	voluntary	black, yellow	thick	no	6	32	35	neck			
Large-spotted genet	*Genetta tigrina*	Viverridae	immobilized	tan	thin	no	6	18	18	abdomen	138	134	stomach
Lesser kudu	*Tragelaphus imberbis*	Bovidae	immobilized	tan	thin	no	4	21	19	abdomen			
Lesser Madagascar hedgehog tenrec	*Echinops telfairi*	Tenrecidae	immobilized	pink	thin	no	7	24	24	chest	108	105.6	stomach
Linne’s two toed sloth	*Choloepus didactylus*	Choloepodidae	voluntary	tan, black	thick	no	8	31	35	nostrils	77	77.4	face
Lion	*Panthera leo*	Felidae	immobilized	tan	thin	no	5	16	15	abdomen, chest	60		
Lion	*Panthera leo*	Felidae	immobilized	tan	thin	no	5	15	17	abdomen, chest	68		
Long-tailed chinchilla	*Chinchilla lanigera*	Chinchillidae	voluntary	gray	thick	no	5	37	49	nostrils	170		
Magellenic penguin	*Spheniscus magellanicus*	Spheniscidae	voluntary	black, white	thick	yes	4						
Masai giraffe	*Giraffa camelopardalis tippelskirchi*	Giraffidae	voluntary	tan	thin	no	6	37	35	abdomen	52	49	inner leg
Minilop rabbit	*Oryctolagus cuniculus*	Leporidae	voluntary	light brown	thick	no	5	41	42	nostrils	219		
Orangutan	*Pongo pygmaeus* x *Pongo abelii*	Hominidae	voluntary	orange	thin	no	7	25	23	nostrils, chest	102	105.6	chest, palm
Plains zebra	*Equus quagga*	Equidae	immobilized	black, white	thin	no	8	19	14	abdomen	43	42	inner leg
Polar bear	*Ursus maritimus*	Ursidae	immobilized	white	thick	yes	8	12	10	abdomen, chest	46	42	groin, snout
Polar bear	*Ursus maritimus*	Ursidae	immobilized	white	thick	yes	7	6	7	abdomen	44	49	groin, mouth
Prehensile-tailed porcupine	*Coendou prehensilis*	Erethizontidae	voluntary	gray	thick	no	5				111		
Radiated tortoise	*Astrochelys radiata*	Testudinidae	voluntary	tan	thick	no	4						
Red footed tortoise	*Chelonoidis carbonarius*	Testudinidae	voluntary	gray	thick	no	6	80	78	neck			
Red-necked wallaby	*Macropus rufogriseus*	Macropodidae	immobilized	light brown	thin	no	7	20	20	chest	108	105.6	ear
Red panda	*Ailurus fulgens*	Ailuridae	voluntary	black	thick	no	7				118	119	inner leg
Red river hog	*Potamochoerus porcus*	Suidae	voluntary	red	thin	yes	4						
Screaming Hairy Armadillo	*Chaetophractus vellerosus*	Chlamyphoridae	voluntary	tan	thick	no	5						
Screaming Hairy Armadillo	*Chaetophractus vellerosus*	Chlamyphoridae	voluntary	tan	thick	no	5						
Slender-tailed meerkat	*Suricata suricatta*	Herpestidae	immobilized	tan, black	thin	no	5				146		
Southern rockhopper penguin	*Eudyptes chrysocome*	Spheniscidae	immobilized	black, white	thick	yes	6	8	7	abdomen			
Southern tamandua	*Tamandua tetradactyla*	Myrmecophagidae	voluntary	black, white	thin	no	6						
Southern three banded armadillo	*Tolypeutes matacus*	Chlamyphoridae	voluntary	tan	thick	no	5						
Tawny frogmouth	*Podargus strigoides*	Podargidae	voluntary	brown	thick	no	6	25.6	26	nostrils	185	133.2	eye
Tawny frogmouth	*Podargus strigoides*	Podargidae	voluntary	brown	thick	no	5						
Virginia opossum	*Didelphis virginiana*	Didelphidae	voluntary	gray	thick	no	5						
White-bearded wildebeest	*Connochaetes taurinus albojubatus*	Bovidae	immobilized	brown	thin	no	6	2	5	chest	71	70.2	inner leg
White-faced Saki monkey	*Pithecia Pithecia*	Pitheciidae	immobilized	black	thin	no	8	15	15	chest	103	106	face
Yellow-backed duiker	*Cephalophus silvicultor*	Bovidae	immobilized	dark brown	thin	no	6	12	10	abdomen	72		

### RGB & IRT video analysis

First, FLIR Research Studio (Teledyne FLIR, Wilsonville, OR) software was used to identify IRT videos of adequate quality to analyze. A scoring system of 0–8 was developed to reflect video quality (see Methods), with 8 being the highest quality videos, so videos of low quality could be excluded from analysis.

**Fig. 1:**
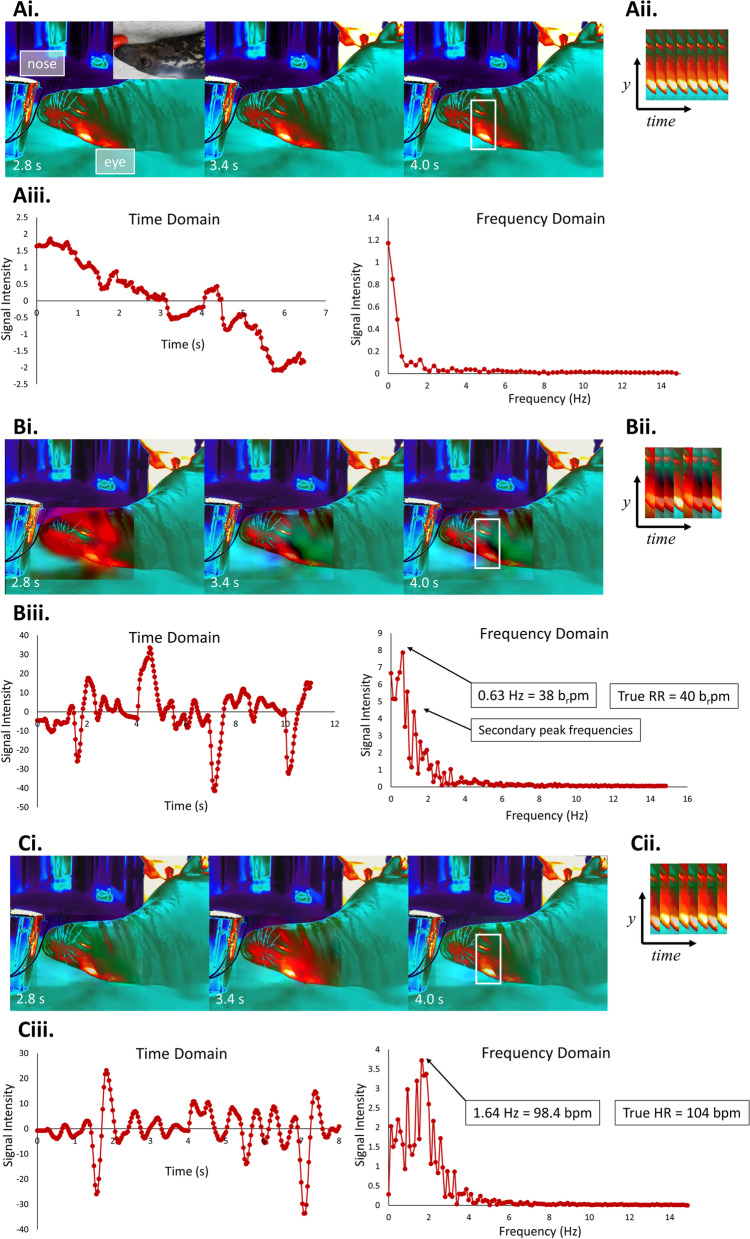
A representative example of Eulerian Video Magnification image processing of a gray seal (*Halichoerus grypus*) infrared video. **Ai.** In the raw infrared video without magnification, there is no visible temporal variation in thermal signatures, as demonstrated by the spatiotemporal slices (**Aii.**). **Aiii.** Signal intensity did not vary over time nor was there a peak frequency intensity. **Bi.** When the infrared video was magnified 40 × with a 0.1–3.5 Hz passband, there was substantial variation in signal intensity through time (**Bii.**). **Biii.** The frequency domain had a clear peak at 0.63 Hz which is assumed to be RR, and 0.63 Hz = 38 breaths per minute (b_r_pm). The ‘true’ RR was 40 b_r_pm. To ensure the narrow passband is not dominated by the RR peak, the narrow passband will be chosen to exclude 0.63 Hz. **Ci.** The infrared video was magnified 40 × with a 1–2 Hz passband, resulting in variation across spatiotemporal slices (**Cii.**), the frequency domain had an obvious peak at 1.64 Hz which is assumed to be HR (**Ciii.**), and 1.64 Hz = 98.4 beats per minute (bpm). The ‘true’ HR via stethoscope was 104 bpm

To magnify small changes in thermal energy associated with blood flow, Eulerian video magnification (EVM) was performed using Lambda Vue (Quanta Computer, Taiwan) software that uses amplification algorithms developed in Wu et al. 2012 and was adapted from Lauridsen et al., 2019 (Fig. 1). Nine second segments of video were used to reduce unmanageable processing, as recommended by Lauridsen et al. 2019. First, a wide passband encompassing 0.1 – 3.5 Hz was used to amplify changes in colored pixels (at 40 × magnification), and extracted signals were normalized. Fourier transformation was used to decompose the signal from each video into its component frequencies. A normalized intensity plot was used to identify the dominant peak intensity, which always corresponded closely with ‘true’ RR, determined by the observation of ribcage expansion and/or nostril flaring from the (RGB) color video.

On the same video, EVM analysis was then repeated. The dominant frequency (taken to be RR) was excluded and a narrower passband ranging 1 Hz in width was used for EVM, to focus on secondary peak intensities. For example, if the dominant peak of the wide passband showed RR was 0.6 Hz, then a narrow passband of 1–2 Hz was used to focus on secondary intensities (as in Fig. 1). The peak frequency from the narrow passband was taken to be HR and was compared to ‘true’ values obtained using a stethoscope (3 M Littmann CORE digital stethoscope, Eko Health, Oakland, CA), ultrasound, manual palpation, or veterinarian’s electrocardiogram (ECG). These true measurements were taken near-simultaneously (within ~ 30 s), as animal movement and other logistics sometimes prevented the stethoscope measurement and infrared imaging to occur at the exact same time.

The analysis workflow developed during this study resulted in two normalized signal intensity plots and peak frequencies (wide passband corresponding with RR, and narrow passband corresponding with HR) for each video (see narrow passband in Fig. 1). An imaging session was considered successful if the video analysis produced a clear peak frequency and that peak frequency was comparable to the ‘true’ measurement of either HR or RR.

### Imaging sessions

‘True’ RR and/or HR were successfully measured in 44 imaging sessions out of 58, which included 44 individuals and 40 species (30 mammals, 6 birds, 4 reptiles) and were used for comparison to IRT-derived measurements. Eighteen of these imaging sessions occurred while the animal was immobilized (45%) and 26 imaging sessions were conducted while the animal voluntarily remained still (65%). Seven individuals were imaged through barriers causing some obstruction via bars or mesh grates, while 37 were imaged with no obstruction.

### Use of infrared thermography versus RGB for vital rate measurements

To identify when IRT was superior to RGB imagery for obtaining vital measurements, EVM analysis was also conducted on recorded color video. A dominant peak for RR could also be extracted using EVM of RGB videos in 27 of the 40 species (67.5%) imaged. Peak frequencies could not be identified after EVM analysis to identify HR in any species using RGB video. This demonstrates that using IRT was necessary to measure animal HR, and this could not be accomplished using RGB video.

### Accuracy and precision of IRT-derived physiological measurements

Non-invasive IRT provided an accurate means with which to measure animal vital rates. Of the 40 different species, broad bandpass frequency EVM analysis of IRT video yielded a prominent peak associated with RR in 36 individuals (81.8%) and 32 species (80%) (see Table 1). This included all species that RR was observed via ribcage expansion or nostril flaring from the RGB videos, and RR could be measured in an additional 5 species with IRT by measuring the change in temperature around the nostrils: Long tailed chinchilla (*Chinchilla lanigera*), harbor seal (*Phoca vitulina*), minilop rabbit (*Oryctolagus cuniculus minilop*), California sea lion (*Zalophus californianus*), Tawny frogmouth (*Podargus strigoides*); see Table 1. Using temperature changes around the nostrils facilitated RR measurements in these additional species, either because animal movement had made it difficult to observe ribcage expansion or the animal had significant subcutaneous fat, fur or plumage. Image analysis provided accurate measurements of RR (from ‘true’ measurements mean absolute error: 1.9 b_r_pm; average percent error: 4.4%), and there was no significant difference between RR values obtained using IRT and ‘true’ RR measurements (*t* = -0.810, *p* = 0.424).

**Fig. 2:**
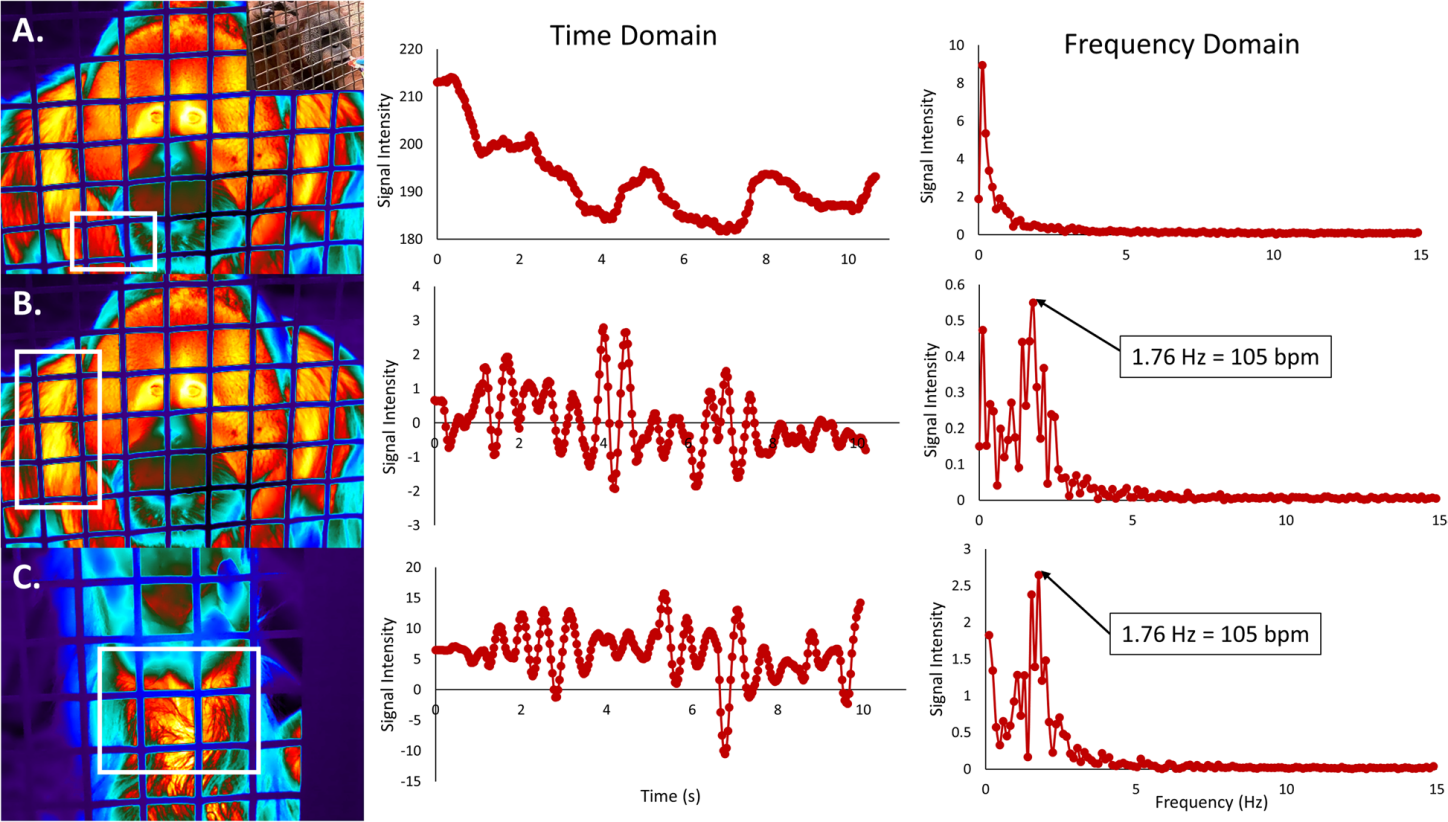
Video analysis outputs from one imaging session of one orangutan (*Pongo pygmaeus* x *Pongo abelii*) focusing on three different locations. All videos were magnified 40 × and had the narrow bandpass (1 – 2 Hz) applied. **A** is the output from analyzing a spot on the chest (marked with the white box) with more fur, which produces no signal. The dominant peak here is probably due to animal movement. The shoulder (**B**) and wrist (**C**) produce the same output, taken to be HR. The stethoscope reading was 102 bpm

In 24 individuals (54.5%) and 22 species (55%), the narrow bandpass frequency analysis yielded a prominent peak representative of HR (see Table 1) and were also highly accurate (from ‘true’ measurements mean absolute error: 2.6 bpm; average percent error: 1.3%); these were statistically indistinguishable from ‘true’ values (*t* = 1.068, *p* = 0.297). The most common point locations on the body with high thermal signatures for HR measurement were temples and inner legs. Figure 2 demonstrates the importance of measuring HR at areas with high thermal signatures.

### Precision of vital rate measurements derived from non-invasive imagery

To demonstrate that the IRT-derived measurements are precise, RR and HR were measured in different parts of the individual in videos from a subset of imaging sessions. RR was measured in more than one location on an animal’s body (nostrils, abdomen, chest) in ten videos and HR was measured in more than one location in seven videos (Tables 2, 3). Vital rate measurements were statistically similar across the body (paired t-test—RR: *t* = 0.190, *p* = 0.8534; HR: *t* = 1.162, *p* = 0.2894; Table 1).

**Table 2:** Successful respiration rate and heart rate measurements differed by sedation status, taxa, integument and fat thickness, and video quality. Percentages refer to successfully extracting a signal for vital rate measurements for that group. Percentages labeled with the same letter are not significantly different from one another, while different letters denote significant differences (*p *< 0.05)

	**Successful RR measurement**	**Successful HR measurement**
**Sedation status**		
Immobilized (*n* = 18)	88.9%^a^	72.2%^a^
Voluntary (*n* = 26)	76.9%^a^	38.5%^b^
**Taxa**		
Mammal (*n* = 33)	84.8%^a^	66.7%^a^
Bird (*n* = 6)	50%^a^	33.3%^b^
Reptile (*n* = 5)	100%^a^	0%^c^
**Thick vs. thin**		
Thick integument (*n* = 21)	66.7%^a^	38.1%^a^
Thin integument (*n* = 23)	95.7%^b^	69.6%^b^
**Quality of video**		
Low quality (≤ 5, *n* = 14)	50%^a^	14.3%^a^
High quality (≥ 6, *n* = 30)	96.7%^b^	74.3%^b^
**Subcutaneous fat**		
Sig. subcutaneous fat (*n* = 10)	60%^a^	40%^a^
No sig. subcutaneous fat (*n* = 34)	88.2%^b^	58.8%^b^

**Table 3:** Best-fit general linear mixed-effect (GLMM) models showing the relationship between ‘true’ and IRT-derived RR and HR values with species ID as a random effect to account for any species where multiple individuals were imaged. Fixed effects were added to investigate the role of physical characteristics in IRT-EVM errors. Models are ordered by AICc, with best models at the top of the table; the base model is included. See Additional file 1, Table S1 for additional model information

**RR models**		
Base model: true RR x IRT RR + species ID (random effect)		
**Added fixed effect**	**AICc**	**R**^**2**^** adjusted**
Thickness of integument	183.17	0.9836
Base model	185.41	0.9647
Immobilized or voluntary	186.47	0.9663
Taxa	189.32	0.9664
Significant presence of subcutaneous fat	187.95	0.9649
Integument	192.40	0.96640
**HR models**		
Base model: true HR x IRT HR + species ID (random effect)		
**Added fixed effect**	**AICc**	**R**^**2**^** adjusted**
Taxa	175.87	0.9573
Integument	179.48	0.9573
Base model	185.00	0.9284
Thickness of integument	187.67	0.9300
Significant presence of subcutaneous fat	187.84	0.9295
Immobilized or voluntary	188.13	0.9287

### Characteristics that make an animal a good candidate for using IRT

Video quality, immobilization status, taxa, thickness of integument, and subcutaneous fat influenced the success of the IRT-derived measurement while animal color, ambient temperature, and humidity did not impact measurements (Table 1). Accurate RR measurements were more robust to animal movement, with no differences in measured RR from immobilized or voluntary animals (${\rm X}$ ^2^ = 1.024, *p* = 0.312), and physical features of the animal (fur, scales, or feathers) (${\rm X}$ ^2^ = 3.902, *p* = 0.142). However, HR measurements (i.e., a peak frequency was identified after EVM analysis) were more likely to be obtained when imagery was collected from immobilized animals (${\rm X}$ ^2^ = 4.860, *p* = 0.027) and from mammals compared to birds and reptiles (${\rm X}$ ^2^ = 6.525, *p* = 0.038).

Successful extraction of physiological signals (RR: ${\rm X}$ ^2^ = 6.200, *p* = 0.013; HR: ${\rm X}$ ^2^ = 4.385, *p* = 0.036) was more likely in animals with thin than thick integument. Similarly, animals without significant subcutaneous fat were more likely to have a successful RR (${\rm X}$ ^2^ = 4.141, *p* = 0.042) and HR validation (${\rm X}$ ^2^ = 25.615,* p* < 0.00001). Video quality also significantly affected the ability to obtain RR (${\rm X}$ ^2^ = 13.974, *p* = 0.0002) and HR measurements (${\rm X}$ ^2^ = 13.424, *p* = 0.0003), with high quality videos (score of 6–8) more likely to produce a clear RR and HR signal.

### Effects of species physical characteristics on accuracy of IRT measurements

**Fig. 3:**
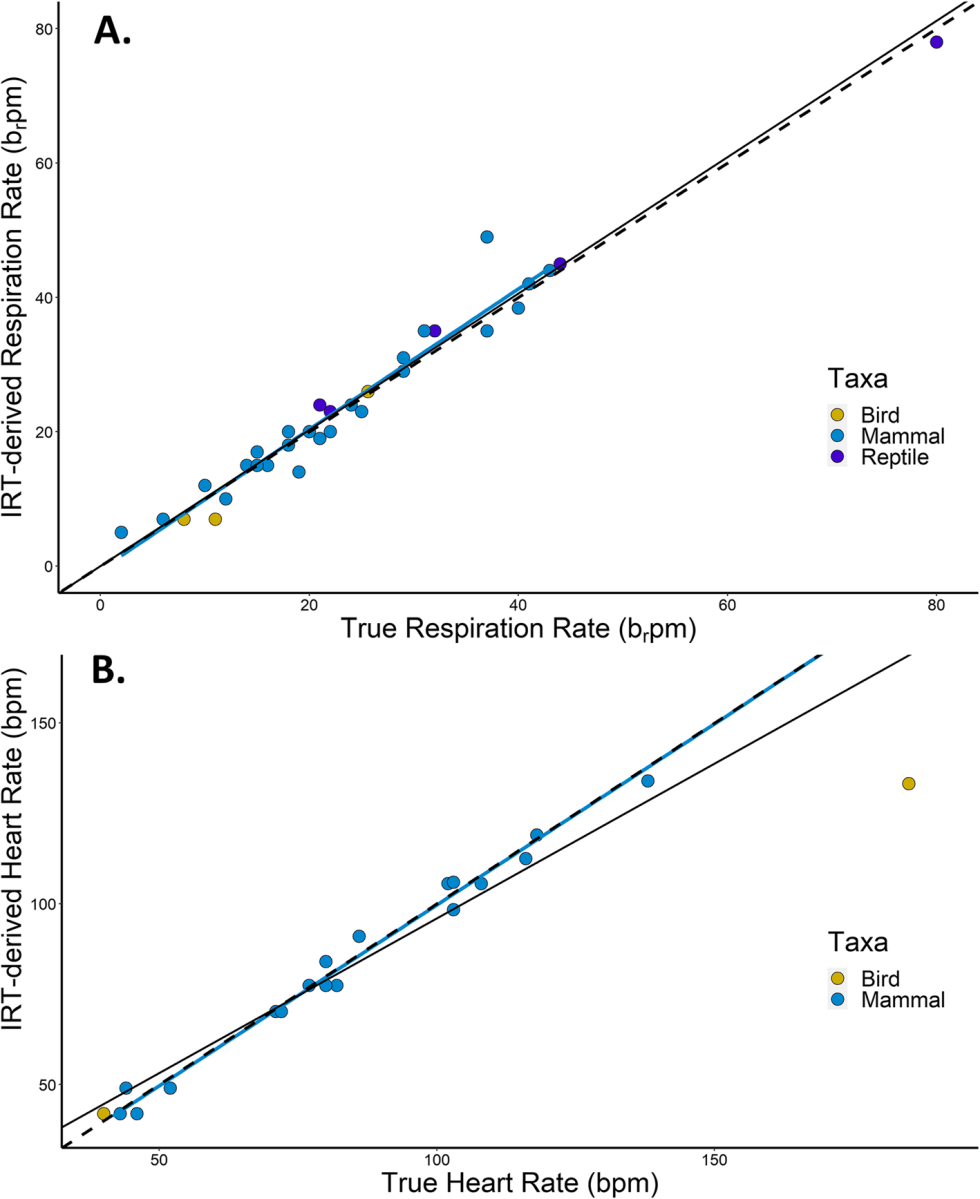
Linear regressions showing the relationship between ‘true’ and IRT-derived **A **respiration rate (RR) and **B** heart rate (HR). Points are color coded by taxa (*blue* = mammal; *yellow* = bird; *purple* = reptile). The dashed black line shows a 1:1 relationship; the solid black regression line shows the relationship between IRT-derived and ‘true’ values for all taxa combined; and the blue regression shows the relationship for mammals only

**Fig. 4:**
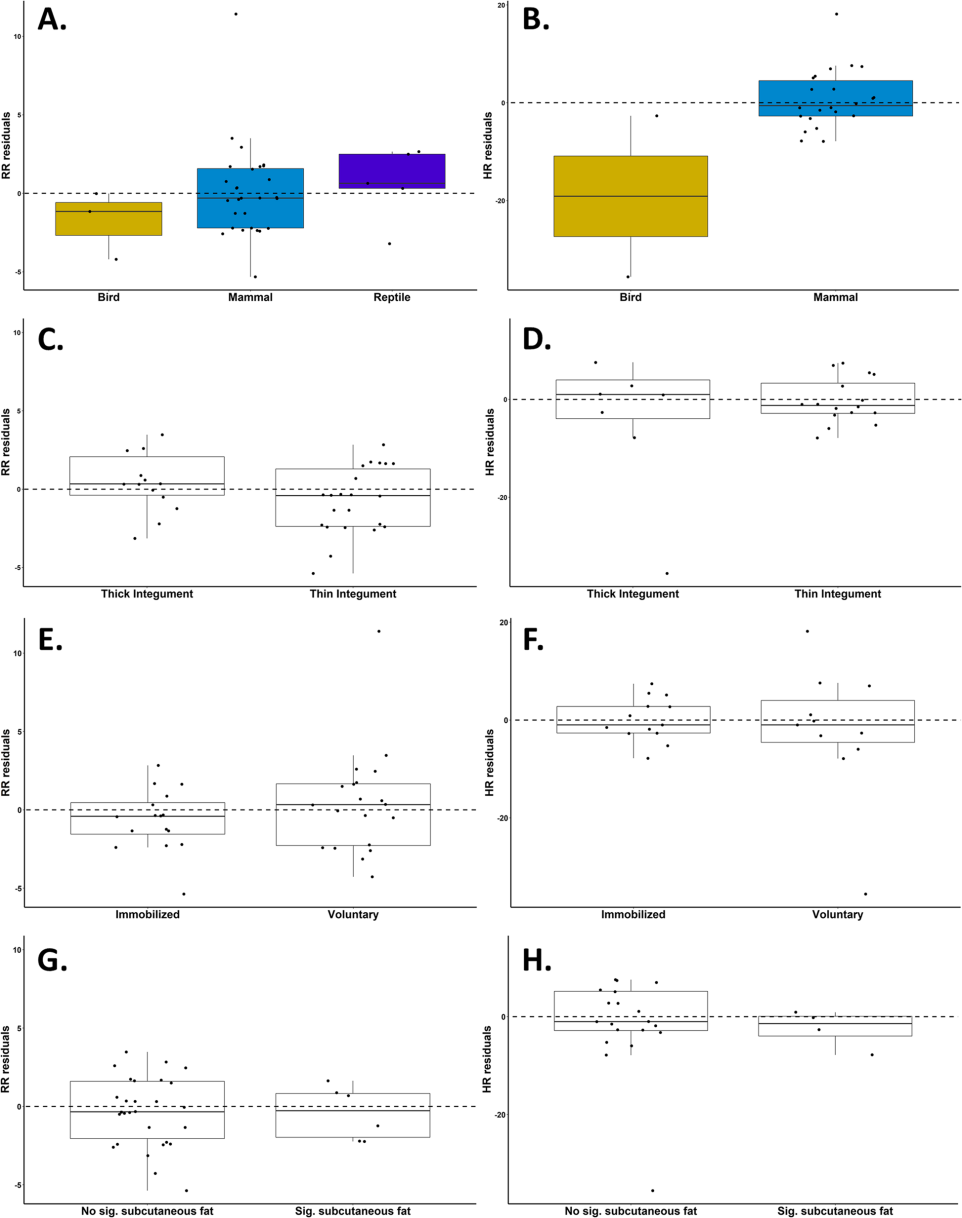
RR and HR residuals by taxa (**A**, **B**), thick or thin integument (**C**, **D**), immobilized or voluntary imaging (**E**, **F**), and presence of significant subcutaneous fat (**G**, **H**). All residuals were taken from the regression lines encompassing all imaging sessions (RR: *y* = 1.0146*x* + 0.0386; HR: *y* = 0.8559*x* + 10.431)

IRT-derived vital rate measurements and ‘true’ values were highly correlated for both RR (Table 3, Fig. 3A; all taxa combined (*n* = 36): *y* = 1.0146*x* + 0.0386, R^2^ = 0.96; mammals only (*n* = 29): *y* = 1.0494*x* – 0.6931; R^2^ = 0.9349) and HR (Table 3, Fig. 3B; all taxa combined (*n* = 25): *y* = 0.856*x* – 10.431, R^2^ = 0.93; mammals only (*n* = 23): *y* = 1.0018*x* – 0.7602, R^2^ = 0.9917), and the slopes did not differ from one. However, some of the species’ physical features contributed to errors in IRT-derived physiological metrics (Fig. 4). The errors in IRT-derived RR relative to ‘true’ RR measurements were higher in animals with thick integument, fur, or scales compared to animals with thinner integument/pelage (Table 3). The accuracy of IRT-derived HR also differed among taxa, with greatest accuracy in mammals (Table 3, Fig. 4).

### Successful vs. failed use of IRT and characteristics that played a part in errors in RR and HR

Because the majority of animals participated voluntarily, not all stayed still long enough to capture videos ≥ 9 s, and as a result IRT did not provide a clear enough signal for RR in 8 of 40 species or HR in 18 of 40 species (Table 4). There were 6 species for which neither IRT-derived RR nor HR could be measured (African crested porcupine (*Hystrix cristata*), prehensile-tailed porcupine (*Coendou prehensilis*), King penguin (*Aptenodytes patagonicus*), blue penguin (*Eudyptula minor*), Magellanic penguin (*Spheniscus magellanicus*), slender-tailed meerkat (*Suricata suricatta*)). These imaging sessions produced videos of low quality due to movement of the animal or background ‘noise’ (i.e., movement) that interfered with EVM analysis and provided no clear peak frequency.

**Table 4:** Failed validations. Species for which there were no peak frequencies extracted related to RR and/or HR are listed here. An ‘X’ in the Validation Failed column indicates which measurement was not obtained via IRT. A suspected reason for failure is listed for each species

Species	Validation Failed		Suspected reason for failure
	**RR**	**HR**	
African crested porcupine	X	X	Too much animal movement
Andean bear	X		Thick fur, face covered by anesthesia mask
Blue penguin	X	X	Too much animal movement, feathers, subcutaneous fat, wet
California sea lion		X	Too much animal movement, wet
Long tailed chinchilla		X	Thick fur, > 1 m from camera
Chuckwalla		X	Too much animal movement, thick scales
Dabb spiny tailed lizard		X	Too much animal movement, thick scales
Dromedary camel		X	Tail movement and moving shadow on thermal windows
Harbor seal		X	Too much animal movement, thick blubber, wet
King penguin	X	X	Too much animal movement, feathers, subcutaneous fat, wet
Magellanic penguin	X	X	Too much animal movement, feathers, subcutaneous fat, wet
Minilop rabbit		X	Thick fur, > 1 m from camera
Prehensile-tailed porcupine	X	X	Too much animal movement, unstable camera
Red footed tortoise		X	Too much animal movement, thick skin/scales
Red panda	X		Too much animal movement
Slender-tailed meerkat	X	X	Bag breathing (immobilized), background movement
Southern rockhopper penguin		X	Feathers, subcutaneous fat (immobilized so not due to movement)

For a subset of animals, ‘true’ HR measurements could not be obtained due to the difficulty of using a stethoscope on animals with thick scales or skin (African elephant (*Loxodonta africana*), hippopotamus (*Hippopotamus amphibius*), gopher tortoise (*Gopherus polyphemus*), ostrich (*Struthio camelus*), radiated tortoise (*Astrochelys radiata*)), eating while imaging (causing the stethoscope to pick up mastication and/or deglutition and not heart rate), or logistical issues in placing the stethoscope on the animal through the enclosure (brown bear (*Ursus arctos*), lesser kudu (*Tragelaphus imberbis*)) (see Additional file 2, Table S2). While these cannot be directly compared to true values, IRT analyses yielded RR and HR values comparable to previous studies in a subset of these animals (Table 5).

**Table 5:** Species for which IRT-derived RR and HR were measured, but no true values could be measured to allow for comparison

Species	IRT RR	RR values from other studies	IRT HR	HR values from other studies
African elephant	10	4 – 12 [30]	28.2	25—30 [31, 32]
Brown bear	12	6 – 10 [33]	60	65 [34] 79 [35]
Lesser kudu	19	21 (from RGB video in this study)	84.6	n/a
Ostrich	14	6 – 12 [36]	84	80 [36]

**Fig. 5:**
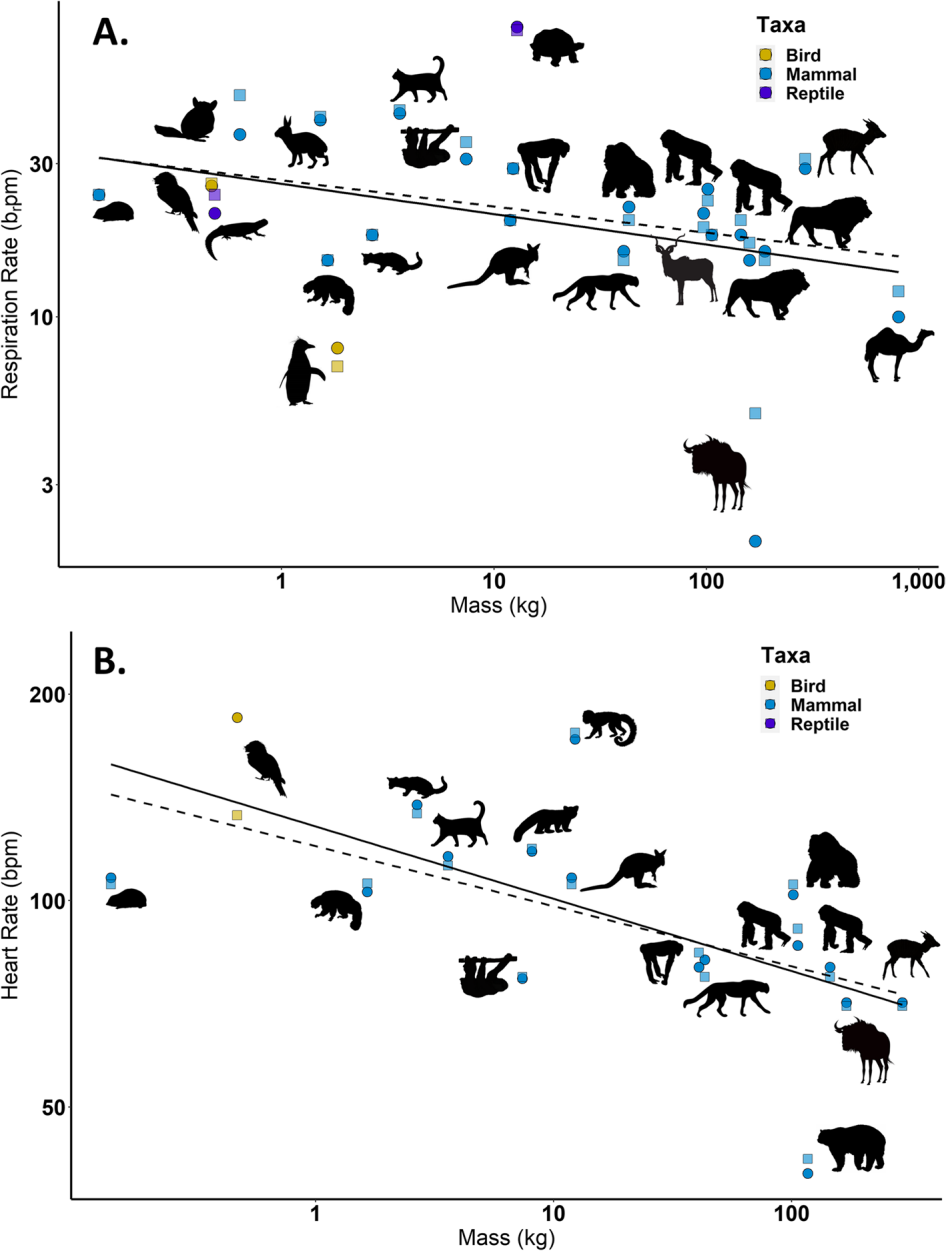
The negative log–log relationship between mass and **A** RR and **B** HR. Squares are ‘true’ values and circles show IRT-derived vital rate values, with all points colored by taxa (*blue* = mammal; *yellow* = bird; *purple* = reptile). The solid black line is the regression of ‘true’ values with body mass, and the dashed black line shows the regression IRT-derived vital rates with body mass

The successful validation of measuring vital rates with IRT allows for its use to measure RR and HR in a range of species, with the potential to address larger ecological questions. For example, the non-invasive IRT-derived measurements had similar relationships with animal body mass, when compared with ‘true’ measurements that required animal training or immobilization, demonstrating the applicability of this method in comparative studies (Fig. 5; A. ‘true’ RR: *y* = -1.659ln(*x*) + 28.749; IRT RR: *y* = -1.916ln(*x*) + 30.323; ‘true’ HR: *y* = -16.4ln(*x*) + 154.32; IRT HR: *y* = -7.907ln(*x*) + 120.82).

## Discussion

This study demonstrates the efficacy of using IRT to obtain metabolic indices in a wide range of species. The validation of new technological and analytical tools (IRT coupled with EVM processing) for accurate vital rate measurements will allow for the expansion of its use in zoo and wildlife studies. When adequate signal intensities could be extracted from IRT videos, these appeared to accurately reflect the animal’s ‘true’ RR (range in error: 0 – 12 brpm) and HR (range in error: 0 – 4.6 bpm; bird outlier: 50 bpm). This demonstrates that while IRT-EVM analysis did not yield adequate signals to measure metabolic indices in every species, it importantly did not ‘pull-out’ false signals.

This study also highlights the characteristics that make an individual or species a good candidate for obtaining IRT-derived measurements. We found that not only did IRT technology provide accurate animal vital rates, but the use of imagery is more time effective, logistically easier, and less invasive than other methods like respirometry or isotope dilution [6, 7]. Most imaging sessions lasted < 3 min, including imaging multiple areas of the body, since only a 9 s video is required to accurately calculate most RR and HR values. Slightly longer videos (15 – 30 s) may be necessary to accurately calculate very low rates. This study identified locations on the body with high thermal signatures, and a priori knowledge the best areas to image (inner leg, temple, groin) would likely shorten imaging times for future applications. Using IRT is logistically simple with limited equipment needed, and because imaging does not require animal immobilization or restraint, it is significantly less invasive than other methods of obtaining metabolic rate, or even just HR.

Because it is non-invasive, IRT-derived RR and HR may also be more likely to be true baseline measurements, whereas handling stress associated with traditional methods are more likely to increase RR and HR [37–39].

The error between ‘true’ and IRT-derived measurements was typically small (< 5%), but there was some variability. This may be the result of changing RR and HR throughout an imaging session, as not all videos and ‘true’ measurements could be taken at the exact same time in every imaging session. Sedatives can also cause abrupt changes in HR [40–42], which may also explain some of the variation in individuals immobilized during imaging. Beyond IRT, it is not uncommon to have variation between methods of obtaining HR, as there have been significant differences between HR via stethoscope and HR via EKG [43]. There can also be slight but non-significant discrepancies between manual and electronic stethoscopes [44]. Some animals in this study did not tolerate the stethoscope, or the stethoscope/ultrasound did not pick up a heartbeat or pulsation through their thick skin or scales. For some of those same individuals, IRT imaging provided a clear dominant peak in a frequency similar to HR measured in previous studies. This suggests that IRT may provide a means to measure HR in animals that will not tolerate applying pressure for stethoscope, EKG reads or palpation, and further highlights the advantage of the non-invasive nature of IRT imaging.

Although some vital rate measurements can be obtained with an RGB camera in humans or animals with no (or limited) pelage [9, 11], this study shows that using infrared thermography makes it possible to measure RR in many species, and was always necessary when measuring HR in exotic species. The IRT determination of RR via change in temperature around the nostrils was often essential when the animal was moving, and it was difficult to distinguish ribcage movement associated with RR. We had no success in extracting peak intensities (assumed to be HR) from RGB GoPro videos. This suggests that applicability of RGB for vital sign measurements may be limited in less controlled settings with voluntary participation of animals in human care or free-ranging animals.

IRT-derived measurements matched ‘true’ measurements in many species, however, there were characteristics of certain animals that made it difficult to successfully obtain their HR and RR. When an imaging session was unsuccessful, it was most likely a combination of animal movement and physical characteristics like subcutaneous fat, scales, or feathers. For this reason, both moving birds and reptiles were more difficult to successfully image compared to moving mammals. Immobilized animals not only had limited movement, but areas with higher thermal signatures such as the stomach and groin were easier to image while the individuals were supine. However, these sites of the body were very difficult to image when the individuals were upright and mobile. Most of the animals that participated voluntarily in this study were receiving positive reinforcement and eating while being imaged, resulting in some movement, especially in the face which often offers the best thermal windows for IRT. If free-ranging animals are imaged while resting or hauled out, movement will be greatly reduced compared to some of the animals in managed care settings.

### Caveats of IRT-derived measurements

While IRT-derived physiological measurements were successfully acquired with low errors in many species, this study also highlights the caveats of this technology. Successful HR measurements were made primarily in mammals, especially those with thin fur or skin, and fewer HR measurements were obtained in reptiles and birds, potentially due to limited ability to detect internal temperature changes in ectotherms [45] or thick plumage in birds [46]. The presence of water/liquid on the animal can also substantially affect the analysis of the infrared video [47] and in the few individuals in this study (California sea lion (*Zalophus californianus*), harbor seal (*Phoca vitulina*), gray seal (*Halichoerus grypus*)) that had recently hauled-out from a pool in their enclosure, it was more difficult to locate a thermal window from which to extract a HR signal. This may limit applicability of the method to terrestrial and/or semi-aquatic animals when hauled-out and dry on land. Additionally, animal movement is a major limitation in the application of IRT-derived RR and HR. Successful RR and especially HR measurements require stillness from the animal, preferably for nine seconds or more. This may make imaging difficult with captive animals that are not trained to remain still and may limit applicability to wild animals when at rest.

Current software allows for real-time visualization of amplified video; however selection of an ROI and extraction of peak frequencies does require user time investment. Video analysis to extract vital rates took approximately 7 min per video in this study.

### Future directions

Further investigation of camera specificity (frames per second, pixels, etc.) to identify the minimum IRT resolution required for EVM analysis in RR and HR measurements may also help make this method more easily accessible if cameras requiring lower financial investment could be utilized. In this study, a standardized distance (~ 1 m) was used to test the feasibility of IRT-EVM analysis. After demonstrated success within close proximity, future studies testing the range and distances from which IRT video can be collected and still yield accurate vital rates would facilitate application of IRT-EVM in less controlled settings such as in the field with free-ranging animals. Both environmental conditions and animal behavior are likely to be more variable and may impact the camera’s ability to detect small fluctuations in temperature. If IRT-derived measurements are accurate in the field, this would greatly broaden the reach of these imaging methods for measurements of metabolic indices in a much larger sample size than if animals had to be restrained or immobilized for physical measurements. That IRT-EVM analysis would provide a powerful tool towards addressing broader ecological questions is also demonstrated by the remarkably similar relationships found between ‘true’ and IRT-derived metabolic indices, with animal mass. Application to large numbers of animals may be simpler if measurements could be made in real time, by leveraging machine learning analysis tools [13]. Along these same lines, if pairing motion tracking software with IRT allowed thermal windows to be tracked while moving, this may help to overcome the observed limitations of animal movement in method application and/or simplify the analysis that is currently done manually [48, 49].

## Conclusions

This is the first study to demonstrate that prominent signals corresponding to RR and HR can successfully be extracted from infrared videos coupled with EVM analysis in a variety of species. The combination of IRT and EVM provides a novel tool for both animal care staff in zoological institutions and wildlife researchers. These results suggest that this approach is best suited for mammals and individuals without thick skin or subcutaneous fat but could be applied to some of these species provided an adequate thermal window was observed. Using IRT to obtain metabolic data in the field and zoo setting is both non-invasive and logistically simple, yielding accurate results while avoiding inherent risks (to researchers and animals) associated with animal capture for traditional hands-on techniques. Many veterinarians and zoos have access to infrared cameras, making IRT-EVM image analysis a promising means to monitor animal health and can be promptly implemented and expanded to additional species. Application of such non-invasive methods can help tailor animal husbandry protocols, and address knowledge gaps in wildlife health that are essential for effective conservation management.

## Methods

### Study animals

Fifty-eight individuals across 52 species (39 mammals, 7 birds, 6 reptiles) were imaged at the Cincinnati Zoo and Botanical Garden in Cincinnati, OH (*n* = 44), the Louisville Zoo in Louisville, KY (*n* = 11), the Columbus Zoo and Aquarium in Columbus, OH (*n* = 2), and the Salisbury Zoo in Salisbury, MD (*n* = 1). Individuals were either immobilized as part of the zoo’s annually scheduled wellness examinations (*n* = 18), or animals voluntarily participated (*n* = 40) through operant conditioning with positive reinforcement (Table 1). Characteristics about the individual (fur length, fur color, integument or blubber thickness, reproductive status, immobilized or imaged voluntarily, mass) and the imaging session (ambient temperature, humidity, through enclosure/barrier or free contact) were recorded to identify if physical or environmental factors impacted the success of obtaining accurate IRT-derived RR and HR.

Fifteen individuals had masses recorded the day of imaging and when a mass estimate wasn’t available, one was used from within 7 days (*n* = 27). All procedures were approved by the Cincinnati Zoo and Botanical Garden and Woods Hole Oceanographic Institution Institutional Animal Care and Use Committees (IACUC) (#21–167; BI25044.03 respectively) and approved by management at collaborating institutions.

### RGB & IRT video analysis

IRT videos were obtained using the FLIR T540 (30 Hz image frequency, 464 × 348 pixel IR resolution with a 24° lens; Teledyne FLIR, Wilsonville, OR) on a tripod. A scoring system was developed to determine video quality. One point was given for each attribute that constituted a high-quality video, for a score up to 8: a steady camera; an area of high thermal signature was visible; little to no movement of the animal; little or no movement in the background; the animal was dry; there were no shadows across the animal; the subject was 1 m (or closer) to the camera; and all those factors are true for ≥ 9 s. Videos with a score of 3 or less were excluded from the analysis. Once videos with a score of 4 or higher were identified, all videos were trimmed to a consistent length of 9 s. This length was chosen to encompass most expected HR and RR frequencies while limiting unmanageable data [11]. The video length was extended to 30 s only if no signal was extracted to ensure very low vital rates could be identified. All IRT videos were exported with a black-and-white color palette for consistency.

A standardized amplification procedure was used to perform Eulerian video magnification (EVM) using Lambda vue (https://lambda.qrilab.com/product/application/, v. 1.0.12, Quanta Computer, Taiwan). For IRT videos, 40 × color magnification was used because pulsation caused a change in temperature, and therefore changes in color. RGB videos were analyzed once with 200 × color magnification and once with 200 × motion magnification to attempt to identify pulsation either by movement within blood vessels or changes in skin color. Higher magnification was used for RGB videos relative to IRT videos because changes in color are more subtle and require more magnification in RGB videos. A region of interest (ROI) was positioned on the area of highest thermal signature to reduce noise from surrounding movement. Two rounds of EVM were applied to each video, with one wide passband and one narrow passband, as described in the results. Frames from the magnified videos were extracted with FFmpeg software and saved as.jpg image sequences. ImageJ 1.8 (National Institutes of Health, USA) was used to extract signals from the frames. The extracted signals were normalized by subtracting the average signal intensity of the entire video clip from the signal intensity at any one time. Fourier transformation was used to decompose the signal from each video into its component frequencies. As explained in the results, dominant peak and secondary peak intensities were identified as RR and HR, respectively.

### Statistics

Analyses were performed in R 4.0.2 using RStudio 1.3 (R Core Team, 2020). True measurements were compared to RGB or IRT-derived measurements using paired two-tailed t-tests. To examine the relationship between true measurements, IRT-derived measurements, and characteristics of the individual, general linear mixed effect models (GLMMs) were run with physical characteristics; taxa, presence of fur, scales, feathers, or skin (integument), fur color, thick integument (defined as > 1 cm) and significant subcutaneous fat (defined as ≥ 2 cm [50, 51], as well as characteristics of the imaging session; if the animal was immobilized or volunteered, ambient temperature, and humidity. To visualize the effect of taxa and physical characteristics on the accuracy of IRT-derived vs. true measurements, residuals from both the RR and HR regressions were compared across taxa, integument, immobilization status, and subcutaneous fat. Because more than one individual was imaged for some species, species ID was included as a random effect. Due to multicollinearity, taxa and the presence of fur, scales, or feathers were never used in the same model. The relationship between a successful imaging session and physical characteristics were determined using chi-squared tests. Results were considered significant at *p* < 0.05, and all models were examined to ensure homoscedasticity.

## Supplementary Information

**Additional file 1: Table S1.** Generalized Linear Models.

**Additional file 2: Table S2.** Unable to get true RR or HR measurements.

## Data Availability

The datasets used and analyzed during the current study are available on Dryad Digital Repository and can be accessed at https://doi.org/10.5061/dryad.r4xgxd2j2. The Lambda vue software used to for Eulerian Video Magnification can be found at https://lambda.qrilab.com/product/application/.

## Abbreviations

IRT: Infrared thermography
EVM: Eulerian video magnification
HR: Heart rate
RR: Respiration rate
RGB: Red-green–blue
ROI: Region of interest
