# Supplementary material for: Non-invasive measurements of respiration and heart rate across wildlife species using Eulerian Video Magnification of infrared thermal imagery
Source: BMC Biol. 2023 Mar 29;21:61. doi: 10.1186/s12915-023-01555-9 (PMC10052854; doi:10.1186/s12915-023-01555-9)
Supplement: Supplementary file 1 — Additional file 1: Table S1. Generalized Linear Models. [file 12915_2023_1555_MOESM1_ESM.docx]

**Supplemental Table 1**: Generalized Linear Models: all included IRT RR x true RR (or IRT HR x true HR) and species as random effect

| **RR models**  **Null model: true RR x IRT RR + species ID** | | | | |
| --- | --- | --- | --- | --- |
| **Added fixed effect** | **AICc** | **Estimate** | ***\| t \|*** | ***p*-value** |
| Thickness of fur/scales/skin/feathers | 183.17 | Thick: 2.49  Thin: -2.46 | Thick: 1.869  Thin: 2.372 | Thick: 0.0721  Thin: 0.025 |
| Null model | 185.41 |  |  |  |
| Immobilized or voluntary | 186.47 | Immobilized: -0.21  Voluntary: 1.33 | Immobilized: 0.231  Voluntary: 1.302 | Immobilized: 0.819  Voluntary: 0.201 |
| Taxa | 189.32 | Mammal: 1.99  Reptile: 2.77  Bird: -1.51 | Mammal: 1.184  Reptile: 1.264  Bird: 0.909 | Mammal: 0.244  Reptile: 0.215  Bird: 0.369 |
| Significant presence of subcutaneous fat | 187.95 | Sig. subcut fat: -0.48  Not sig. subcut fat:  0.19 | Sig. subcut fat: 0.373  Not sig. subcut fat: 0.189 | Sig. subcut fat: 0.712  Not sig. subcut fat: 0.851 |
| Fur, feathers, scales, or skin | 192.40 | Fur: 2.00  Feathers: -1.52  Scales: 2.76  Skin: 1.54 | Fur: 1.192  Feathers: 0.911  Scales: 1.262  Skin: 0.487 | Fur: 0.241  Feathers: 0.368  Scales: 0.215  Skin: 0.629 |

| **HR models** |  |  |  |  |
| --- | --- | --- | --- | --- |
| **Added fixed effect** | **AICc** | **Estimate** | ***\| t \|*** | **p-value** |
| Taxa | 175.87 | Mammal: 21.61  Bird: -11.97 | Mammal: 4.020  Bird: 1.788 | Mammal: 0.0005  Bird: 0.086 |
| Fur, feathers, or skin | 179.48 | Fur: 21.58  Feathers: -11.94  Skin: 21.98 | Fur: 4.002  Feathers: 1.778  Skin: 2.509 | Fur: 0.0005  Feathers: 0.089  Skin: 0.019 |
| N/A (base model) | 185.00 |  |  |  |
| Thickness of fur/scales/skin/feathers | 187.67 | Thick: 7.73  Thin: 3.04 | Thick: 1.301  Thin: 0.753 | Thick: 0.205  Thin: 0.458 |
| Significant presence of subcutaneous fat | 187.84 | Sig. subcut fat: -3.37  Not sig. subcut fat: 12.05 | Sig. subcut fat: 0.625  Not sig. subcut fat: 2.222 | Sig. subcut fat: 0.538  Not sig. subcut fat: 0.036 |
| Immobilized or voluntary | 188.13 | Immobilized: 10.66  Voluntary: -1.25 | Immobilized: 2.201  Voluntary: 0.320 | Immobilized: 0.573  Voluntary: 0.752 |
