# Supplementary material for: Non-invasive measurements of respiration and heart rate across wildlife species using Eulerian Video Magnification of infrared thermal imagery
Source: BMC Biol. 2023 Mar 29;21:61. doi: 10.1186/s12915-023-01555-9 (PMC10052854; doi:10.1186/s12915-023-01555-9)
Supplement: Supplementary file 2 — Additional file 2: Table S2. Unable to get true RR or HR measurements. [file 12915_2023_1555_MOESM2_ESM.docx]

**Supplemental Table 2**: Unable to get true RR or HR measurements

| **Species** | **Suspected reason for failure** |
| --- | --- |
| African elephant | Stethoscope did not work on thick skin |
| Barn owl | Did not tolerate stethoscope, animal movement |
| Bat-eared fox | Too much movement (eating during imaging) so could not detect RR or HR |
| Brown bear | Hard to place stethoscope on animal through grate in enclosure |
| Central American tapir | Hard to access animal around enclosure, lots of movement |
| Gopher tortoise | Stethoscope did not work though shell; animal did not tolerate stethoscope on neck |
| Hippopotamus | Thick skin- stethoscope did not work |
| Lesser kudu | Did not tolerate stethoscope, lots of movement |
| Ostrich | Stethoscope did not work on thick skin, too much animal movement |
| Radiated tortoise | Stethoscope did not work through shell; animal did not tolerate stethoscope on neck |
| Red river hog | Too much movement |
| Screaming hairy armadillo | Too much movement (eating during imaging) so could not detect RR or HR |
| Southern tamandua | Too much movement (eating during imaging) so could not detect RR or HR |
| Southern three banded armadillo | Too much movement (eating during imaging) so could not detect RR or HR |
| Virginia opossum | Too much movement (eating during imaging) so could not detect RR or HR |
